# Current status and influencing factors of decision conflict among surrogate decision-makers for patients with severe traumatic brain injury in the ICU

**DOI:** 10.3389/fmed.2026.1892560

**Published:** 2026-08-12

**Authors:** Simiao Wang, Jun Li, Xiaohui Zhang, Wei Xu, Yi Li

**Affiliations:** 1 Department of Critical Care Medicine, Surgery Section, The First Affiliated Hospital of Henan University of Science and Technology, Luoyang, China; 2 Department of Nursing, First Affiliated Hospital of Henan University of Science and Technology, Luoyang, China; 3 Department of Gastrointestinal Surgery, The First Affiliated Hospital of Henan University of Science and Technology, Luoyang, China; 4 Henan University of Science and Technology, Luoyang, China

**Keywords:** clinical decision-making, critical care nursing, decision conflict, influencing factors analysis, severe traumatic brain injury, surrogate decision-maker

## Abstract

**Objective:**

To investigate the current status of decision conflict among surrogate decision-makers for patients with severe traumatic brain injury in the intensive care unit (ICU) and to analyze its influencing factors.

**Methods:**

From January to June 2025, a convenience sampling method was used to select 148 surrogate decision-makers of patients with severe traumatic brain injury admitted to a tertiary A-level hospital in Henan Province as the study participants. Data were collected using a general information questionnaire, the Decision Conflict Scale, the Decision Preparedness Scale, and the Shared Decision-Making Questionnaire.

**Results:**

The Decision Conflict Scale score among surrogate decision-makers of ICU patients with severe traumatic brain injury was 43.36 ± 10.23. Overall, 92.6% of surrogate decision-makers experienced decision conflict, with 61.5% reporting high levels of decision conflict. Decision conflict levels were negatively correlated with decision preparedness and participation in shared decision-making (*r* = −0.403, −0.381; *P* < 0.05). Multiple linear regression analysis showed that family per capita monthly income, decision preparedness, and level of participation in shared decision-making were influencing factors of decision conflict among surrogate decision-makers.

**Conclusion:**

Surrogate decision-makers for ICU patients with severe traumatic brain injury experience moderate to high levels of decision conflict. Lower family per capita monthly income, lower decision preparedness, and lower participation in shared decision-making among surrogate decision-makers are associated with higher levels of decision conflict. Enhancing decision preparedness and promoting the implementation of shared decision-making can help reduce decision conflict among surrogate decision-makers.

## Introduction

Traumatic brain injury (TBI) represents a major public health challenge in China. Epidemiological data indicate that the annual incidence of TBI is approximately 55 ∼ 64 per 100,000 population, of which severe traumatic brain injury (STBI) accounts for 21.4%. The disability rate among patients with STBI is as high as 45% and continues to increase annually, making it one of the most common conditions encountered in surgical intensive care units (1). Following injury, most patients with STBI fall into a comatose state and lose autonomous decision-making capacity, requiring surrogate decision-makers to make medical decisions on their behalf.

The critical and complex clinical conditions of patients with severe traumatic brain injury (STBI) involve numerous medical decision-making nodes with trade-offs among multiple therapeutic options, resulting in high complexity in clinical decision-making. For instance, decompressive craniectomy can reduce mortality and shorten the length of ICU stay in STBI patients (2, 3). However, postoperative skull defects may trigger a series of complications such as intracranial hemorrhage and hydrocephalus, and this surgical approach fails to significantly improve long-term neurological prognoses, including persistent vegetative state and severe disability (4, 5). Furthermore, the high uncertainty of therapeutic outcomes further complicates clinical decision-making. Relevant studies have demonstrated that approximately 80% of STBI patients die after the withdrawal of life-sustaining treatment, while a considerable proportion of patients remain in a vegetative state even after active intensive treatment, creating a critical dilemma in choosing between active rescue treatment and palliative care (6). Additionally, STBI patients are unable to express their personal treatment preferences due to persistent disturbance of consciousness. Meanwhile, the advance directive system has not been widely implemented in China, making patients’ true wills inaccessible for clinical decision-making (7, 8). Most surrogate decision-makers make medical choices based on their personal emotions and values rather than the authentic preferences of patients. Accordingly, surrogate decision-makers for STBI patients in the ICU face multiple decision-making dilemmas, including urgent decision time limits, coexisting therapeutic risks and benefits, uncertain treatment outcomes, and a lack of reference for patients’ genuine intentions. Currently, professional decision-making support in ICU settings is insufficient, which frequently leads to decision conflict among surrogate decision-makers, defined as a state of uncertainty when confronted with multiple competing therapeutic choices (9). Epidemiological data indicate that the incidence of decision conflict among ICU surrogate decision-makers is as high as 63.5% (10). Decision conflict severely impairs the physical and mental health of surrogate decision-makers and increases their risk of developing anxiety, depression, and other psychological disorders (11). Moreover, decision conflict may reduce the quality of clinical decisions, trigger decision delays, hinder the progression of clinical treatment, and ultimately adversely affect patients’ therapeutic outcomes (12).

Previous studies on decision conflict among surrogate decision-makers have primarily focused on populations with chronic diseases (13), cancer (14), and general ICU patients (15). However, surrogate decision-makers of patients with STBI in the ICU represent a distinct population with unique characteristics, and the level and influencing factors of decision conflict in this group may differ substantially from those in other populations. Therefore, investigating the current status and influencing factors of decision conflict among surrogate decision-makers of patients with STBI in the ICU has important clinical implications.

The Ottawa Decision Support Framework proposes that individuals experience different types of decision needs and that decision needs and decision outcomes interact with one another. Adequate decision support can simultaneously influence decision needs and decision outcomes (16). Guided by this framework, the present study hypothesized that decision preparedness (decision needs) and shared decision-making (decision support) among surrogate decision-makers of patients with STBI may both influence decision conflict (decision outcome). Mature and validated questionnaires and scales were selected based on a literature review to explore the factors influencing decision conflict in this population, with the aim of providing evidence for the development of targeted intervention strategies in the future.

## Materials and methods

### Study design and participants

A convenience sampling method was used to recruit surrogate decision-makers of patients with severe traumatic brain injury who were admitted to the ICU of a tertiary Grade A hospital in China between January 2025 and June 2025. Inclusion criteria for patients were as follows: (1) age ≥18 years; (2) meeting the diagnostic criteria for severe traumatic brain injury (17); (3) meeting ICU admission criteria, with first-time ICU admission and an ICU stay of ≥3 days (18); (4) lack of autonomous decision-making capacity as determined by the attending physician (e.g., impaired consciousness, cognitive dysfunction, or inability to communicate verbally, physically, or in writing). Exclusion criteria for patients were as follows: (1) withdrawal of life-sustaining treatment during hospitalization; (2) incomplete clinical data; (3) concurrent participation in other research studies. Inclusion criteria for surrogate decision-makers were as follows: (1) age ≥18 years; (2) primary responsibility for signing informed consent forms for the patient’s medical treatment. Exclusion criteria for surrogate decision-makers were as follows: (1) inability to cooperate with the survey due to emotional distress; (2) a history of mental disorders or current use of psychotropic medication or receipt of psychological treatment.

The sample size was estimated based on the rule-of-thumb criterion for multiple linear regression. A total of 20 independent variables were incorporated into the regression model, consisting of three categories of indicators: 11 dummy categorical variables extracted from general information (gender, educational level, relationship with the patient, marital status, and per capita monthly household income), 3 continuous demographic variables (surrogate decision-maker age, patient age, and ICU length of stay), and 6 standardized continuous scale scores from three measurement instruments, including the total score of the Shared Decision-Making Questionnaire, the standardized score of the Decision Readiness Scale, as well as three dimensional subscores and one total score of the Decisional Conflict Scale. Consistent with the methodological standards for quantitative nursing research, multiple linear regression analysis requires 5–10 participants per independent variable (19), generating an initial sample size ranging from 100 to 200 participants. A 20% attrition allowance was set to offset invalid questionnaires and loss to follow-up, resulting in an adjusted target sample size of 126–252 participants. Finally, 148 surrogate decision-makers of ICU patients with severe traumatic brain injury were enrolled, and this sample size satisfied the statistical requirements for multiple linear regression analysis. This study was approved by the Ethics Committee of the participating hospital (approval number: 2024-1755). All participants provided written informed consent and took part in the survey voluntarily.

## Measures

### General information questionnaire

A self-designed general information questionnaire was developed based on clinical experience and relevant literature. Patient variables included age and length of ICU stay. Surrogate decision-maker variables included age, gender, educational level, relationship to the patient, marital status, and average monthly household income per capita.

### Shared Decision-Making Questionnaire

The Shared Decision-Making Questionnaire was developed by Kriston et al. (20) and translated into Chinese by Luo et al. (21). The questionnaire consists of nine items within a single dimension and is rated on a 6-point Likert scale ranging from 0 (“completely disagree”) to 5 (“completely agree”). The raw total score ranges from 0 to 45 and is converted to a standardized percentage score, with higher scores indicating greater perceived involvement and willingness to participate in shared decision-making. The Cronbach’s α of the original scale was 0.945, and the Cronbach’s α in the present study was 0.926.

### Preparation for Decision Making Scale

The Preparation for Decision Making Scale (Prep DMS) was developed by Bennett et al. (22) and translated into Chinese by Li (23). The scale contains 10 items in a single dimension and is rated on a 5-point Likert scale from 1 (“not at all”) to 5 (“a great deal”). Higher scores indicate greater preparedness to participate in treatment decision-making. After conversion to standardized scores, a total score ≥60 indicates good decision preparedness, whereas a score <60 indicates poor preparedness. The original Cronbach’s α was 0.946, and the Cronbach’s α in this study was 0.713.

### Decision Conflict Scale

The Decision Conflict Scale (DCS) was developed by O’Connor et al. (24) and translated into Chinese by Wang (25). The scale comprises 16 items across three dimensions: information and values, perceived effectiveness of decision-making, and decision uncertainty. Items are rated on a 5-point Likert scale from 0 (“strongly agree”) to 4 (“strongly disagree”), with total scores ranging from 0 to 100. Scores <25 indicate no decision conflict, scores of 25 ∼ 37.5 indicate moderate decision conflict, and scores >37.5 indicate high decision conflict. Higher scores represent greater decision conflict. The original Cronbach’s α was 0.886, and the Cronbach’s α in the present study was 0.847.

### Data collection

Data were collected by two investigators who received standardized training prior to the study. Face-to-face questionnaire surveys were conducted using uniform instructions. Before data collection, investigators explained the study objectives, procedures, and relevant considerations to surrogate decision-makers and confirmed that they had not participated in other questionnaire-based studies. After written informed consent was obtained, questionnaires were administered on site. Patient demographic and clinical data were extracted from medical records, while surrogate decision-makers completed their own demographic information and all questionnaire items independently. Upon completion, questionnaires were reviewed by investigators for missing data, which were completed immediately when necessary. A total of 155 questionnaires were distributed, and 148 valid questionnaires were returned, resulting in an effective response rate of 95.5%.

### Statistical methods

Statistical analyses were performed via SPSS 27.0. Categorical variables were described as frequencies and constituent ratios; all continuous variables were subjected to normality tests, with normally distributed data expressed as mean ± standard deviation and compared using independent-samples *t*-test and one-way ANOVA. Pearson correlation analysis was used to explore correlations between decision conflict of surrogate decision-makers for ICU patients with severe traumatic brain injury, shared decision-making and decision preparation. The standardized decision conflict score was set as the dependent variable, and univariately significant variables were entered into multivariate linear regression to identify independent predictors. To quantify the unique contribution of each predictor and enrich analytical dimensions, two variance decomposition indicators were adopted to decompose model total R^2^: standardized gression coefficient β for cross-variable comparison of standardized effect sizes, and squared semipartial correlation (sr^2^) to calculate the unique explained outcome variance of each variable after adjusting other confounders. Variance inflation factor (VIF) was computed to detect multicollinearity, and a two-sided α = 0.05 was defined as the threshold for statistical significance (*P* < 0.05).

## Results

### General characteristics of participants

The age of the 148 surrogate decision-makers ranged from 27 to 67 years, with a mean of 44.60 ± 11.47 years. The patients’ ages ranged from 22 to 80 years, with a mean of 55.40 ± 13.34 years. The length of ICU stay ranged from 3 to 15 days, with a mean of 5.79 ± 2.77 days. Other general characteristics are presented in Table 1.

**TABLE 1:** Comparison of decision conflict scores among surrogate decision-makers with different characteristics (*n* = 148).

Variable	*n* (%)	Decision conflict score (mean ± SD)	Test statistic	*P*-value
Gender			*t* = −0.497	0.620
Male	81 (54.7)	42.98 ± 10.18		
Female	67 (45.3)	43.82 ± 10.34		
Relationship to patient				
Child	75 (50.7)	41.65 ± 10.23	*F* = 1.495	0.219
Spouse	42 (28.4)	44.94 ± 9.73		
Parent	20 (13.5)	44.84 ± 10.79		
Other	11 (7.4)	46.31 ± 10.49		
Marital status			*F* = 0.588	0.556
Married	131 (88.5)	43.32 ± 10.23		
Unmarried	12 (8.1)	41.93 ± 11.26		
Other	5 (3.4)	47.81 ± 8.24		
Average monthly household income per capita (RMB)				
<3000	43 (29.1)	48.80 ± 7.83	*F* = 12.893	<0.001
3000 ∼ 6000	69 (46.6)	42.75 ± 9.60		
>6000	36 (24.3)	38.02 ± 10.97		
Educational level			*F* = 6.094	<0.001
Junior high school or below	48 (32.4)	48.24 ± 7.93		
Senior high school/technical secondary school	33 (22.3)	41.81 ± 10.38		
Junior college	28 (18.9)	41.07 ± 10.17		
Bachelor’s degree or above	39 (26.4)	40.31 ± 10.84		

*t*-values were used for two-group comparisons; *F*-values were used for comparisons among multiple groups.

### Status of decision conflict among surrogate decision-makers

The mean decision conflict (DC) score among surrogate decision-makers was 27.75 ± 6.55, corresponding to a standardized score of 43.36 ± 10.23. Among the three dimensions of the Decision Conflict Scale, the uncertainty dimension had the highest mean score (1.89 ± 0.56), followed by the factors influencing decision-making dimension (1.85 ± 0.65), while the effective decision-making dimension had the lowest mean score (1.34 ± 0.42). Of the 148 surrogate decision-makers, 11 (7.4%) reported no decision conflict, whereas 137 (92.6%) experienced decision conflict. Specifically, 34 (24.8%) had a moderate level of decision conflict, and 91 (61.5%) had a high level of decision conflict.

### Comparison of decision conflict scores across different characteristics

Univariate analysis showed that average monthly household income per capita and educational level of surrogate decision-makers were significantly associated with decision conflict scores (*P* < 0.05). No significant differences were observed for sex, relationship to the patient, or marital status (Table 1).

### Correlation between decision conflict, continuous baseline information and independent variables

Pearson correlation analysis showed that surrogate decision-maker age was significantly positively correlated with decisional conflict (*r* = 0.281, *P* < 0.01). ICU length of stay (*r* = −0.069, *P* > 0.05) and patient age (*r* = −0.034, *P* > 0.05) had no significant correlation with decisional conflict. The mean score of the Shared Decision-Making Questionnaire among surrogate decision-makers was 66.71 ± 16.45. Decision conflict scores were negatively correlated with shared decision-making scores (*r* = −0.403, *P* < 0.01). The mean score of the Preparation for Decision Making Scale among surrogate decision-makers was 52.64 ± 7.58. Decision conflict scores were also negatively correlated with decision preparedness scores (*r* = −0.381, *P* < 0.01). Detailed results are presented in Table 2.

**TABLE 2:** Pearson correlation matrix of decisional conflict, independent variables and continuous.

Variable	ICU length of stay	Surrogate decision-maker age	Patient age	Standardized score of Preparation for Decision Making Scale	Total score of Shared Decision-Making Questionnaire
Surrogate decision-maker age	0.122	−0.078	0.044	0.249**	−0.403**
Patient age	0.181*				
Standardized score of Preparation for Decision Making Scale	−0.034	−0.220**			
Total score of Shared Decision-Making Questionnaire	0.029	−0.250**	−0.028		
Standardized score of Decisional Conflict Scale	−0.069	0.281**	−0.034	−0.381**	

*P < 0.05;

**P < 0.01.

### Multivariate linear regression analysis of decision conflict

Variance inflation factors (VIFs) were calculated to evaluate multicollinearity across all predictors; a threshold of VIF > 10 was adopted to identify severe multicollinearity (26). The VIF values of all independent variables ranged from 1.074 to 1.166, all below 2.0, which suggested no obvious multicollinearity within the regression model. All variables with statistical significance in univariate analyses were simultaneously entered into the multivariate linear regression model using the enter method, and the coding rules for independent variables are presented in Table 3. The overall regression model was statistically significant (*F* = 11.675, *P* < 0.001), with *R*^2^ = 0.291 and adjusted *R*^2^ = 0.266, indicating that the included predictors collectively explained 26.6% of the total variance in decision conflict. Standardized regression coefficient β and squared semipartial correlation (*sr*^2^) were jointly applied to quantify the independent predictive strength and unique explained variance of each variable. As shown in Table 4, average monthly household income per capita (β = −0.284, *sr*^2^ = 0.024, *P* = 0.029), decision preparedness standardized score (β = −0.249, *sr*^2^ = 0.048, *P* = 0.001) and total shared decision-making score (β = −0.314, *sr*^2^ = 0.067, *P* < 0.001) were three independent protective factors for decision conflict among surrogate decision-makers. Higher monthly household income, better decision preparation and higher shared decision-making levels were associated with lower decision conflict. Educational level and surrogate decision-maker age exhibited no significant independent predictive effect on decision conflict (*P* > 0.05), and their unique explained variance *sr*^2^ was close to 0, representing negligible contribution to the outcome variance. Among all significant predictors, shared decision-making possessed the largest absolute standardized β and the highest *sr*^2^ value, demonstrating the dominant unique contribution to decision conflict variation.

**TABLE 3:** Assignment of independent variables.

Independent variable	Coding
Educational level	Junior high school or below (Z1 = 0, Z2 = 0, Z3 = 0); Senior high school/technical secondary school (Z1 = 1, Z2 = 0, Z3 = 0); Junior college (Z1 = 0, Z2 = 1, Z3 = 0); Bachelor’s degree or above (Z1 = 0, Z2 = 0, Z3 = 1)
Average monthly household income per capita (RMB)	<3000 (Z_1_ = 0, Z_2_ = 0), 3000 ∼ 6000 = (Z_1_ = 1, Z_2_ = 0), >6000 = (Z_1_ = 0, Z_2_ = 1)
Surrogate decision-maker age	Continuous variable
Decision preparedness (standardized score)	Continuous variable
Shared decision-making (total score)	Continuous variable

**TABLE 4:** Multivariate linear regression analysis of decision conflict among surrogate decision-makers (*n* = 148).

Variable	Unstandardized β	SE	Standardized β ’	*t*	*P*	*sr* ^2^
Constant	75.049	5.579		13.451	<0.001	
Educational level	0.176	1.208	0.021	0.146	0.884	0.000
Average monthly household income per capita (RMB)	−3.972	1.801	−0.284	−2.205	0.029	0.024
Surrogate decision-maker age	−0.044	0.130	−0.050	−0.342	0.733	0.000
Decision preparedness (standardized score)	−0.336	0.104	−0.249	−3.239	0.001	0.048
Shared decision-making (total score)	−0.435	0.102	−0.314	−4.264	<0.001	0.067

*R*^2^ = 0.291; adjusted *R*^2^ = 0.266; *F* = 11.675; *P* < 0.001.

## Discussion

The overall level of decision conflict among surrogate decision-makers of ICU patients with severe traumatic brain injury was relatively high in this study. This level was higher than that reported by Miller et al. (27) and Jia Xiuli et al. (8). One possible explanation is the lack of a well-established decision support system in Chinese ICUs. On the one hand, various forms of decision support are widely implemented in ICUs in other countries, such as family-centered rounds, decision aids, and regular family meetings. These strategies help alleviate psychological stress and enhance decision participation among surrogate decision-makers (28–30). In contrast, related practices in China are still in an exploratory stage. On the other hand, STBI is characterized by sudden onset. Surrogate decision-makers are often required to make multiple high-risk decisions within a short period and without adequate preparation, which increases the likelihood of decision conflict (31). In this study, decision conflict among surrogate decision-makers was mainly driven by uncertainty, particularly regarding disease-related information, the benefits and risks of treatment options, and clarification of patient values. This may be associated with limited communication time in the ICU, information asymmetry between healthcare providers and families, and the absence of structured decision support mechanisms. These findings suggest that ICU clinicians should pay close attention to the complexity of decision-making situations faced by surrogate decision-makers of patients with STBI, systematically assess the sources of decision conflict, and strengthen decision support.

This study found that household monthly per capita income was negatively correlated with the level of decision conflict among surrogate decision-makers of ICU patients with severe traumatic brain injury. This finding differs from the results reported by Qiao Xiaoting et al. (32) and Sun et al. (33), which may be attributable to differences in disease context. In the present study, most patients with STBI sustained injuries as a result of traffic accidents and faced issues such as liability determination, advance payment of high medical expenses, and limited insurance reimbursement during the early stage of treatment. Low-income families bear greater financial pressure, which may exacerbate decision-making difficulties. Moreover, individuals from low-income households often have limited access to information regarding traffic accident compensation, medical assistance programs, and legal consultation (34), resulting in uncertainty about potential financial reimbursement and further intensifying decision conflict. Given the high uncertainty of prognosis in STBI (35), even patients who survive the acute phase often require long-term rehabilitation. For low-income families, the need to consider their ability to afford subsequent treatment costs may lead to pronounced conflict over whether to continue aggressive treatment. Therefore, clinical practice should emphasize interdepartmental collaboration, promote coordination between hospitals, traffic authorities, and medical assistance funds, establish pre-review pathways for financial assistance for economically disadvantaged families, and simplify application procedures to reduce early financial burden. Nurses can also provide education on traffic accident–related medical assistance policies to mitigate decision conflict arising from information asymmetry.

The results of this study indicated that higher levels of decision preparedness were associated with lower levels of decision conflict among surrogate decision-makers of patients with STBI in the ICU, which is consistent with previous findings (36, 37). In this study, the decision preparedness of surrogate decision-makers was at a low-to-moderate level and similar to the findings reported by Wang Lumeng et al. (38). This suggests that existing health education materials and decision support approaches may be insufficient to adequately prepare surrogate decision-makers for decision-making. One possible reason is the manner and content of information delivery. Currently, decision-related information is primarily communicated verbally by healthcare providers and is often highly summarized (39). Although traditional informed consent forms contain treatment-related information, they rely heavily on text and medical terminology, making them difficult to understand within a short time frame. As a result, surrogate decision-makers may struggle to fully comprehend the disease status and the risks and benefits of treatment options, thereby limiting their level of preparedness (40, 41). Therefore, future clinical practice should focus on addressing gaps in decision preparedness by optimizing how decision information is presented. Key treatment options, comparisons of risks and benefits, and expected outcomes could be conveyed through visual aids, structured question-and-answer formats, and concise decision booklets (paper-based or electronic). Decision guidance and explanations provided before and after key decision points may further enhance decision preparedness and reduce decision conflict.

This study demonstrated that higher levels of shared decision-making participation were associated with lower decision conflict among surrogate decision-makers, consistent with findings reported by Sun Xucan et al. (42). In this study, the level of shared decision-making participation among surrogate decision-makers was moderate, which was consistent with the findings reported by Lu Qing et al. (43). Many surrogate decision-makers perceived that their preferences for decision participation were insufficiently explored during physician–family communication. This may be due to limited awareness of shared decision-making among ICU healthcare professionals and a lack of proactive inquiry into surrogate decision-makers’ desired level of involvement. In addition, surrogate decision-makers under high stress may perceive themselves as lacking sufficient medical knowledge or competence to participate in decision-making and may therefore defer responsibility to physicians, further constraining the shared decision-making process (39, 44). These findings highlight the importance of promoting shared decision-making practices in the ICU to reduce decision conflict. Future efforts should focus on strengthening shared decision-making training for ICU healthcare professionals, actively assessing surrogate decision-makers’ preferences for participation, clearly defining the boundaries of decision-making responsibilities between clinicians and families, and enhancing information exchange and value clarification. Such approaches can support patient-centered decision-making, facilitate shared decision-making, and ultimately reduce decision conflict.

## Research limitations

This study has several limitations. First, this study adopted a single-center convenience sampling method, which resulted in selection bias and limited the sample representativeness and generalizability of the research conclusions. Future studies can adopt a multicenter sampling design to further improve and verify the research findings. Second, the sample size of this study was determined by the classical empirical method for cross-sectional studies without standardized *a priori* statistical power analysis. Although the empirical method is suitable for descriptive cross-sectional studies with good feasibility, it lacks standardized quantitative verification and reduces the methodological rigor of the research to a certain extent. Future studies can conduct *a priori* power analysis using statistical software such as G*Power to accurately estimate the sample size and improve the scientificity and standardization of study design. Third, the Cronbach’s α coefficient of the Preparation for Decision Making Scale in this study was 0.713, which was at the lower limit of the acceptable range and might slightly affect the stability of measurement results. Future studies can select evaluation tools with better reliability and validity and optimize scale items through pre-investigation before formal data collection to further improve data quality and the reliability of research results.

## Conclusion

This study found that surrogate decision-makers of ICU patients with severe traumatic brain injury generally experienced moderate to high levels of decision conflict. The main influencing factors included household monthly per capita income, shared decision-making, and decision preparedness. Healthcare professionals should develop individualized and targeted interventions based on these influencing factors to reduce decision conflict, improve decision quality, and ultimately optimize patient outcomes.

## Data Availability

The raw data supporting the conclusions of this article will be made available by the authors, without undue reservation.
